# Modern Management of Asymptomatic Carotid Stenosis: A Meta‐Analysis of CREST‐2, SPACE‐2, and ECST‐2

**DOI:** 10.1002/acn3.70479

**Published:** 2026-07-13

**Authors:** Aasim Ali, Waqar Ahmed Cheema, Fiza Nisar, Muhammad Shamoon, Sameen Sarfaraz, Ahmad Butt, Muhammad Abdullah Ali, Armaghana Abdullah, Muhammad Asad Shabbir, Muhammad Talha, Hammad Azam, Aimen Anwaar Pannun, Usama Saleem, Anousha Tanveer, Mukesh Sharma

**Affiliations:** ^1^ Neurology Department Allied Hospital Faisalabad Faisalabad Pakistan; ^2^ Internal Medicine Department Allied Hospital Faisalabad Faisalabad Pakistan; ^3^ Neurology Department Dhankuta District Hospital Dhankuta Nepal

**Keywords:** asymptomatic carotid stenosis, carotid revascularization, contemporary medical therapy, meta‐analysis, randomized controlled trials

## Abstract

**Background:**

Recent randomized trials (CREST‐2, SPACE‐2, and ECST‐2) have compared carotid revascularization (carotid endarterectomy [CEA] or carotid artery stenting [CAS]) plus contemporary medical therapy (CMT) versus CMT alone in asymptomatic carotid stenosis. ECST‐2 included both asymptomatic and low‐risk symptomatic patients, introducing clinical heterogeneity. Individual trials reported low event rates and limited statistical power.

**Objective:**

To evaluate whether carotid revascularization reduces long‐term ipsilateral stroke (> 30 days) compared with CMT alone and to assess periprocedural complications.

**Methods:**

Phase 3/4 randomized controlled trials published after 2020 were included. The primary outcome was long‐term ipsilateral stroke excluding periprocedural events. Periprocedural complications (30‐day stroke, myocardial infarction, or death) were analyzed separately. Random‐effects models were used. Subgroup analyses by modality (CEA vs. CAS) included CREST‐2 and SPACE‐2. Sensitivity analyses excluding ECST‐2 and using split‐control methodology for SPACE‐2 were performed.

**Results:**

Three trials (5 arms; 3438 patients) were included. Median follow‐up was 4.0 years in CREST‐2, 5.0 years in SPACE‐2, and 2.0 years in ECST‐2. In the primary pooled analysis including ECST‐2, revascularization showed a nonsignificant reduction in long‐term ipsilateral stroke (RR 0.51; 95% CI 0.21–1.29; *p* = 0.16; *I* ^2^ = 70%) and a borderline increase in periprocedural complications (RR 2.78; *p* = 0.06). Sensitivity analyses excluding ECST‐2 suggested reduced long‐term ipsilateral stroke with revascularization (RR 0.35; 95% CI 0.21–0.58; *p* < 0.0001; *I* ^2^ = 0%; ARR 2.74%; NNT = 37) but increased periprocedural complications (RR 4.54; *p* = 0.004; *I* ^2^ = 0%; ARI 1.45%; NNH = 69). Subgroup analyses suggested a possible benefit with CAS, whereas CEA showed a nonsignificant trend; however, subgroup analyses were underpowered. Split‐control sensitivity analyses for SPACE‐2 yielded directionally similar findings with wider confidence intervals.

**Conclusion:**

The primary pooled analysis was inconclusive and demonstrated substantial heterogeneity. Sensitivity analyses restricted to purely asymptomatic populations suggested that carotid revascularization may reduce long‐term ipsilateral stroke but increase periprocedural complications. Contemporary medical therapy remains the foundation of management for asymptomatic carotid stenosis, while revascularization should be reserved for carefully selected patients after individualized risk assessment and shared decision‐making.

**Abbreviations**
ACAS: Asymptomatic Carotid Atherosclerosis Study
ACST: Asymptomatic Carotid Surgery Trial
CAS: carotid artery stenting
CEA: carotid endarterectomy
CI: confidence interval
CMT: Contemporary Medical Therapy
CREST‐2: carotid revascularization and medical management for asymptomatic carotid stenosis trial
ECST‐2: European Carotid Surgery Trial 2
GRADE: grading of recommendations assessment, development and evaluation
*I* ^2^
: *I*‐squared statistic
MI: myocardial infarction
NNH: number needed to harm
NNT: number needed to treat
PRISMA: Preferred Reporting Items for Systematic Reviews and Meta‐Analyses
RCT: Randomized Controlled Trial
RoB: risk of bias
RR: risk ratio
SPACE‐2: Stent‐Protected Angioplasty versus Carotid Endarterectomy in Asymptomatic Patients

## 1 Introduction

The optimal management of asymptomatic carotid stenosis (ACS) remains controversial despite decades of investigation [1, 2]. Earlier landmark randomized trials, including the Asymptomatic Carotid Atherosclerosis Study (ACAS) and the Asymptomatic Carotid Surgery Trial (ACST), demonstrated that carotid endarterectomy (CEA) reduced the long‐term risk of ipsilateral stroke compared with medical therapy alone [3, 4]. However, these studies were conducted during an era in which medical management was substantially less intensive than current standards. Lipid‐lowering therapy was inconsistently used, high‐intensity statins were not routinely prescribed, and structured vascular risk‐factor modification strategies were limited [5, 6]. Consequently, the absolute benefit observed with revascularization in historical trials may not accurately reflect outcomes achievable in the modern era of optimized medical therapy.

Over the past two decades, contemporary medical therapy (CMT) has evolved considerably and now includes high‐intensity statins, aggressive blood pressure control, antiplatelet therapy, smoking cessation, diabetes management, and structured lifestyle modification [7, 8]. These advances have substantially lowered the annual risk of ipsilateral stroke in medically treated patients with ACS, with several contemporary cohorts reporting annual stroke rates below 1% [9]. As a result, the incremental benefit of carotid revascularization observed in historical surgical trials may be attenuated in current clinical practice [10, 11]. This evolving risk–benefit balance has renewed uncertainty regarding the role of routine carotid intervention in asymptomatic patients and highlights the importance of reassessing revascularization strategies in the context of modern optimized medical therapy.

In response, three major randomized controlled trials were designed to reassess the role of revascularization in the modern era: CREST‐2 (Carotid Revascularization and Medical Management for Asymptomatic Carotid Stenosis Trial), SPACE‐2 (Stent‐Protected Angioplasty versus Carotid Endarterectomy in Asymptomatic Patients), and ECST‐2 (European Carotid Surgery Trial 2) [12, 13, 14]. Each trial individually faced challenges including slow recruitment, lower‐than‐expected event rates, and inconclusive results [15, 16]. Importantly, ECST‐2 included a mixed population of asymptomatic and low‐to‐intermediate risk symptomatic patients, introducing potential heterogeneity that may affect pooled estimates [14].

Given their conceptual alignment but methodological differences, a pooled analysis confined to these modern trials offers the statistical power necessary to resolve the clinical question for contemporary practice [11, 17]. This meta‐analysis addresses the critical question: after accounting for procedural risk, does revascularization provide durable long‐term stroke prevention benefit beyond what is achieved with modern medical therapy alone, and how does the inclusion of ECST‐2's mixed population affect the results? [18, 19].

## 2 Methods

### 2.1 Study Selection

This meta‐analysis was conducted in accordance with the Preferred Reporting Items for Systematic Reviews and Meta‐Analyses (PRISMA) guidelines. We included phase 3 or 4 randomized controlled trials that met the following criteria: randomization of patients with asymptomatic carotid stenosis to contemporary medical therapy (CMT) alone versus CMT plus carotid revascularization (CEA or CAS); protocol‐mandated high‐intensity statin therapy in > 70% of patients and guideline‐directed antiplatelet therapy; full manuscript publication on or after January 1, 2020; reporting of both periprocedural events (within 30 days) and long‐term ipsilateral stroke outcomes separately; and minimum follow‐up of 2 years. ECST‐2 was included despite its mixed population, with prespecified sensitivity analysis excluding this trial to assess its impact on pooled estimates.

Trials published prior to 2020 were excluded because they utilized medical therapy that did not include high‐intensity statins as a protocol‐mandated component, resulting in substantially higher background stroke risk (2.0%–3.2% annually) compared to modern trials (< 1% annually).

A systematic search of MEDLINE, Embase, and the Cochrane Central Register of Controlled Trials was performed from January 1, 2015, to December 31, 2025, using search terms: “asymptomatic carotid stenosis,” “randomized controlled trial,” “carotid endarterectomy,” “carotid stenting,” and “medical therapy (Figure S1 shows full search strategy).”

### 2.2 Outcomes

The primary outcome was long‐term ipsilateral ischemic stroke occurring after the periprocedural period (from 30 days postrandomization through the end of follow‐up). The secondary outcome was periprocedural complications: composite of stroke, myocardial infarction, and death occurring within 30 days of randomization (or within 30 days of revascularization for the intervention arm). Subgroup analysis by revascularization modality (CEA vs. CAS) was prespecified.

### 2.3 Data Extraction and Quality Assessment

**FIGURE 1:**
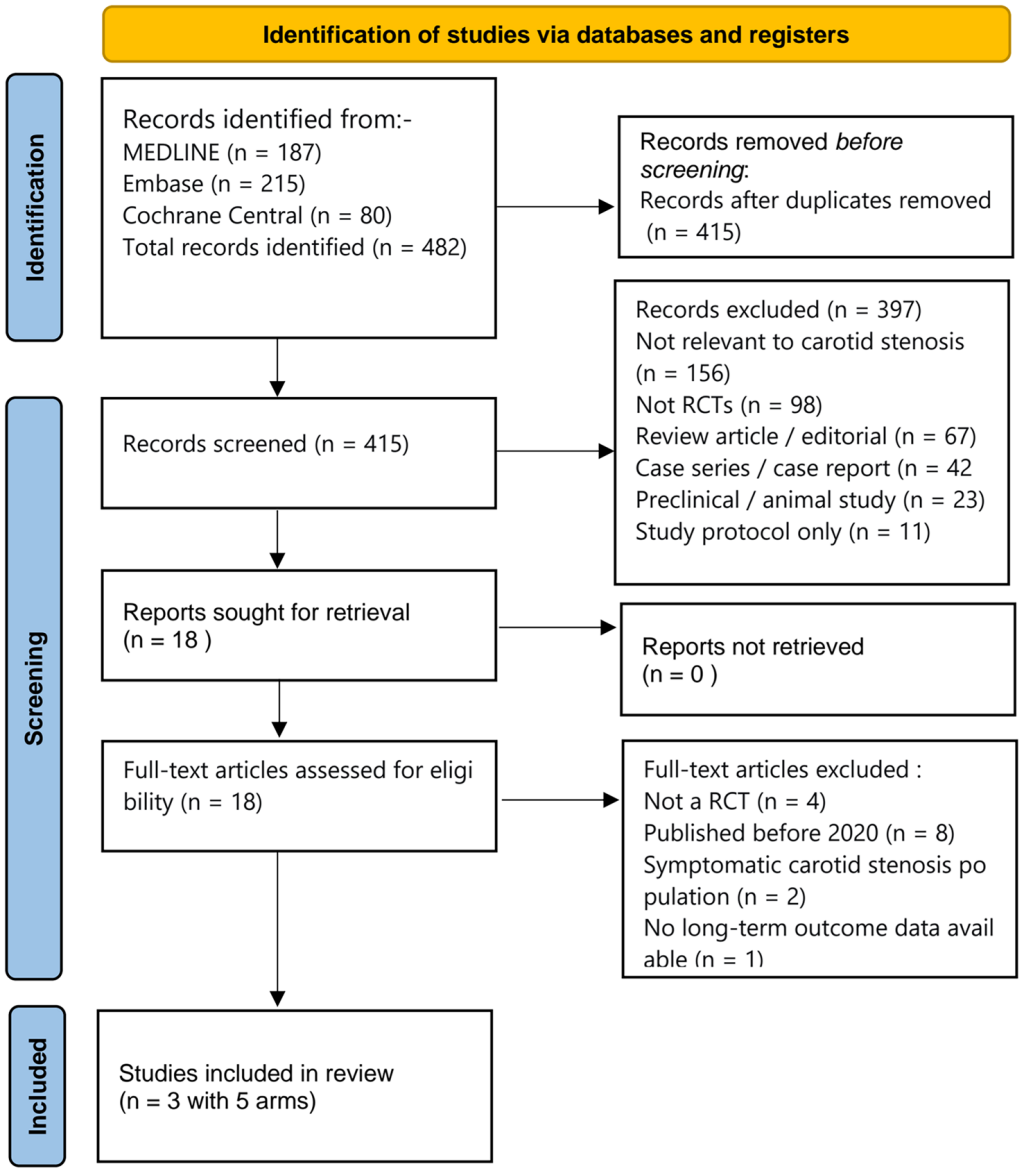
PRISMA flow chart of included studies. *Source*: Page etal. [20]. This work is licensed under CC BY 4.0. To view a copy of this license, visit https://creativecommons.org/licenses/by/4.0.

**FIGURE 2:**
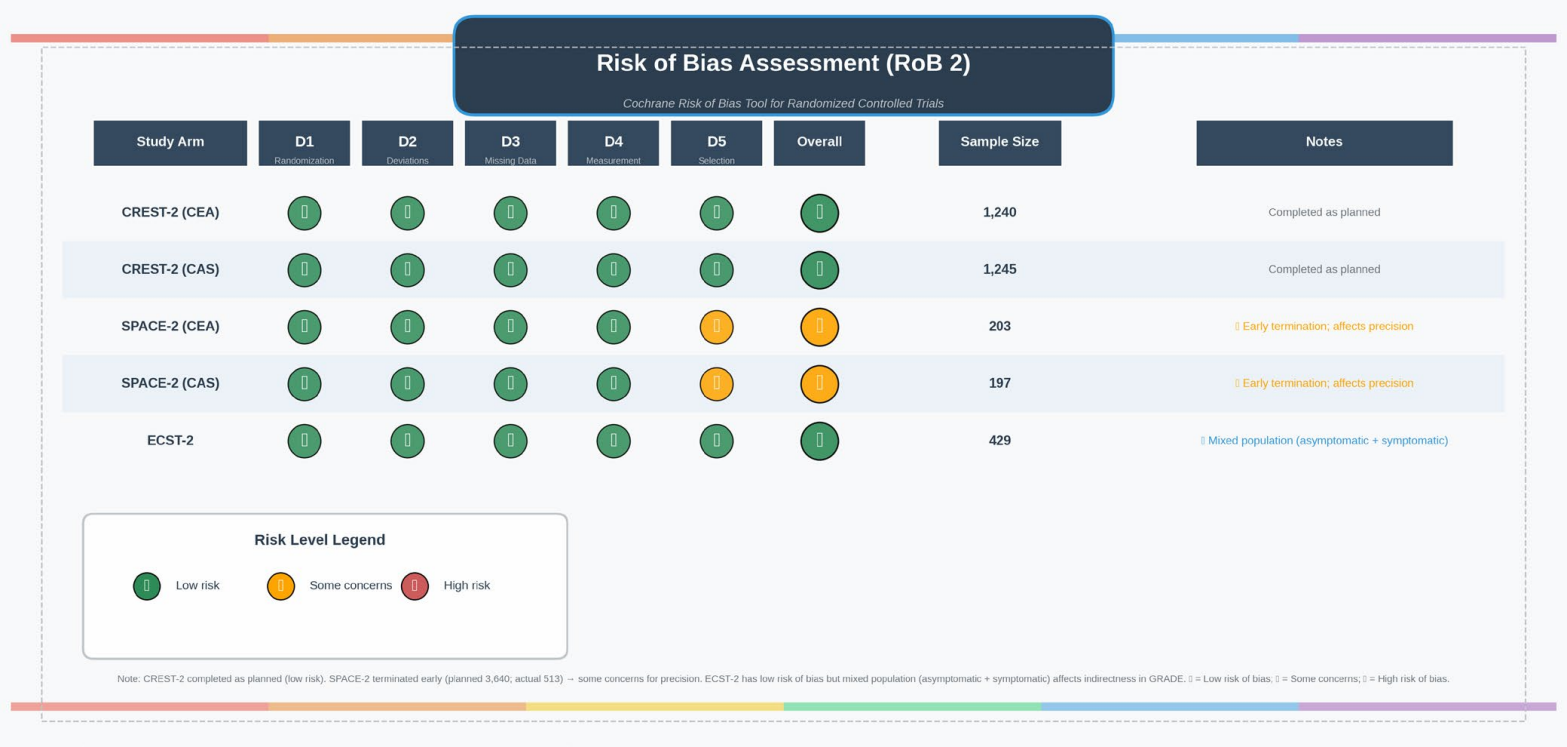
Risk of Bias Assessment (RoB 2). Risk of bias assessment using the Cochrane RoB 2 tool for five study arms from three trials (CREST‐2, SPACE‐2, ECST‐2). Green circle = low risk; Amber circle = some concerns; Red circle = high risk. CREST‐2 completed as planned (low risk). SPACE‐2 terminated early → some concerns for precision. ECST‐2 low risk but mixed population affects indirectness. Sample sizes (*N*) are shown. CAS, carotid artery stenting; CEA, carotid endarterectomy; RoB, risk of bias.

Two independent reviewers extracted data on study characteristics, patient demographics, stenosis severity, revascularization modality, and outcome events. Disagreements were resolved by consensus (Figure 1 shows PRISMA FLOW diagram). Risk of bias was assessed using the Cochrane Risk of Bias Tool for Randomized Trials (RoB 2), shown in Figure 2.

For SPACE‐2, which had two intervention arms (CEA and CAS) sharing a single control arm, the same control group was used for both comparisons without splitting, as this approach maintains the original randomization structure and is consistent with recent meta‐analyses in this field. In addition, sensitivity analyses using split‐control methodology in accordance with Cochrane recommendations were performed and uploaded as (Figure S1). The direction of effect remained unchanged, although confidence intervals widened and statistical precision was reduced.

### 2.4 Statistical Analysis

All analyses were conducted on an intention‐to‐treat basis. Risk ratios (RRs) with 95% confidence intervals (CIs) were calculated for each trial using the Mantel–Haenszel method. A random‐effects model (DerSimonian‐Laird) was prespecified as the primary analysis to account for anticipated clinical and methodological heterogeneity across trials. Heterogeneity was assessed using the *I* ^2^ statistic. Sensitivity analyses were performed using a fixed‐effects model and by excluding ECST‐2 to assess the impact of its mixed population on pooled estimates. Subgroup analyses were performed by revascularization modality (CEA vs. CAS) with tests for subgroup differences. All analyses were performed using RevMan Web (Cochrane Collaboration).

## 3 Results

### 3.1 Study Selection and Characteristics

The systematic search yielded 482 records. After screening, three trials met inclusion criteria: CREST‐2 (with separate randomized comparisons for CEA vs. CMT and CAS vs. CMT), SPACE‐2 (with separate CEA and CAS arms sharing a single control group), and ECST‐2 (single combined revascularization arm). The combined sample size was 3438 patients. Baseline characteristics were well‐balanced across trials (Table 1). Contemporary medical therapy was robust in all trials, with high‐intensity statin utilization exceeding 85% in the CMT arms.

**TABLE 1:** Characteristics of included studies.

Study arm	Trial	Publication year	*N*	Stenosis threshold	Revascularization modality	Follow‐up duration	Periprocedural definition
1	CREST‐2 (CEA arm)	2025	1240	≥ 70% (US)	CEA	4.0 years (median)	30‐day stroke, death
2	CREST‐2 (CAS arm)	2025	125	≥ 70% (US)	CAS	4.0 years (median)	30‐day stroke, death
3	SPACE‐2 (CEA arm)	2021	203	≥ 70% (ECST)	CEA	5.0 year^a^	30‐day stroke, death
4	SPACE‐2 (CAS arm)	2021	197	≥ 70% (ECST)	CAS	5.0 year^a^	30‐day stroke, death
5	ECST‐2	2025	429	≥ 50% (US)	Combined CEA/CAS	2.0 years (median)	30‐day stroke, MI, death

^a^ ECST‐2 included mixed asymptomatic and low‐risk symptomatic patients.

### 3.2 Main Analysis (Including ECST‐2)

#### 3.2.1 Primary Outcome: Long‐Term Ipsilateral Stroke

**FIGURE 3:**
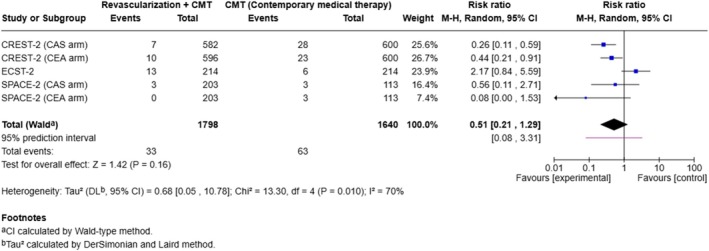
Forest Plot—Long‐term Ipsilateral Stroke (Including ECST‐2): Forest plot of long‐term ipsilateral stroke (excluding periprocedural events) comparing carotid revascularization plus contemporary medical therapy (CMT) versus CMT alone, including ECST‐2. Data from five study arms across three trials: Carotid Revascularization and Medical Management for Asymptomatic Carotid Stenosis Trial (CREST‐2), Stent‐Protected Angioplasty versus Carotid Endarterectomy in Asymptomatic Patients (SPACE‐2), and European Carotid Surgery Trial 2 (ECST‐2). The pooled risk ratio (RR) was 0.51 (95% CI 0.21–1.29; *p* = 0.16), with substantial heterogeneity (*I* ^2^ = 70%), driven by ECST‐2 which demonstrated an opposite direction of effect (RR 2.17). Squares represent study‐specific RRs with size proportional to weight; horizontal lines represent 95% confidence intervals; diamond represents pooled estimate.

Among patients who survived the periprocedural period free of stroke, revascularization plus CMT was associated with a nonsignificant reduction in subsequent ipsilateral stroke compared to CMT alone (RR 0.51; 95% CI 0.21–1.29; *p* = 0.16; *I* ^2^ = 70%). Significant heterogeneity was present, driven largely by ECST‐2, which demonstrated an opposite direction of effect (RR 2.17; 95% CI 0.84–5.59) (Figure 3).

### 3.3 Sensitivity Analysis (Excluding ECST‐2)

**FIGURE 4:**
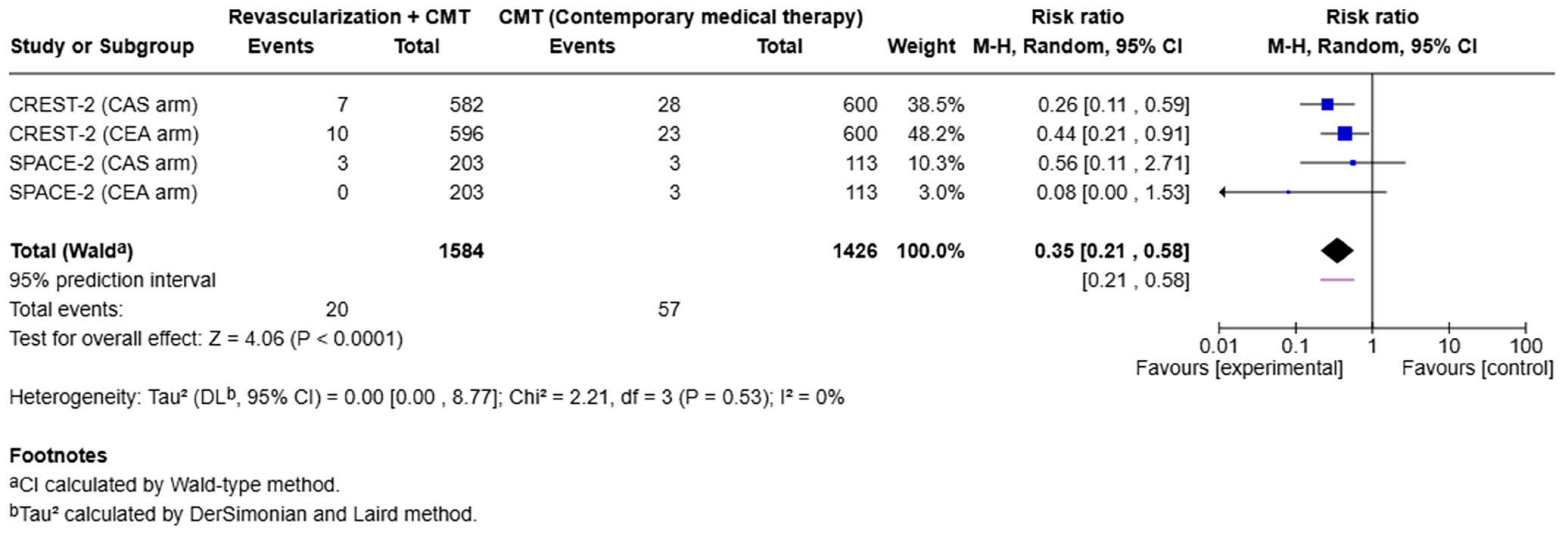
Forest Plot—Long‐term Ipsilateral Stroke (Excluding ECST‐2—Sensitivity Analysis). Forest plot of long‐term ipsilateral stroke (excluding periprocedural events) comparing carotid revascularization plus contemporary medical therapy (CMT) versus CMT alone, excluding ECST‐2 (sensitivity analysis). Data from four study arms across two trials: Carotid Revascularization and Medical Management for Asymptomatic Carotid Stenosis Trial (CREST‐2) and Stent‐Protected Angioplasty versus Carotid Endarterectomy in Asymptomatic Patients (SPACE‐2). The pooled risk ratio (RR) was 0.35 (95% CI 0.21–0.58; *p* < 0.0001), with no heterogeneity (*I* ^2^ = 0%). Squares represent study‐specific RRs with size proportional to weight; horizontal lines represent 95% confidence intervals; diamond represents pooled estimate.

After excluding ECST‐2 (which included a mixed asymptomatic and symptomatic population), revascularization was associated with a significant reduction in long‐term ipsilateral stroke (RR 0.35; 95% CI 0.21–0.58; *p* < 0.0001; *I* ^2^ = 0%) (Figure 4). The absolute risk reduction was 2.74% (NNT = 37).

#### 3.3.1 Secondary Outcome: Periprocedural Complications

##### 3.3.1.1 Main Analysis (Including ECST‐2)

**FIGURE 5:**
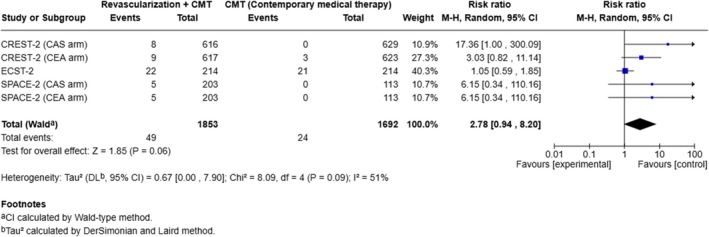
Forest Plot—Periprocedural Complications (Including ECST‐2). Forest plot of periprocedural complications (30‐day stroke, myocardial infarction, or death) comparing carotid revascularization plus contemporary medical therapy (CMT) versus CMT alone, including ECST‐2. Data from five study arms across three trials: Carotid Revascularization and Medical Management for Asymptomatic Carotid Stenosis Trial (CREST‐2), Stent‐Protected Angioplasty versus Carotid Endarterectomy in Asymptomatic Patients (SPACE‐2), and European Carotid Surgery Trial 2 (ECST‐2). The pooled risk ratio (RR) was 2.78 (95% CI 0.94–8.20; *p* = 0.06), with moderate heterogeneity (*I* ^2^ = 51%), driven by ECST‐2 which showed no increased risk (RR 1.05). Squares represent study‐specific RRs with size proportional to weight; horizontal lines represent 95% confidence intervals; diamond represents pooled estimate.

Revascularization was associated with a borderline nonsignificant increase in periprocedural complications compared to CMT alone (RR 2.78; 95% CI 0.94–8.20; *p* = 0.06; *I* ^2^ = 51%). ECST‐2 showed no increased risk (RR 1.05; 95% CI 0.59–1.85), contributing to heterogeneity (Figure 5).

##### 3.3.1.2 Sensitivity Analysis (Excluding ECST‐2)

**FIGURE 6:**
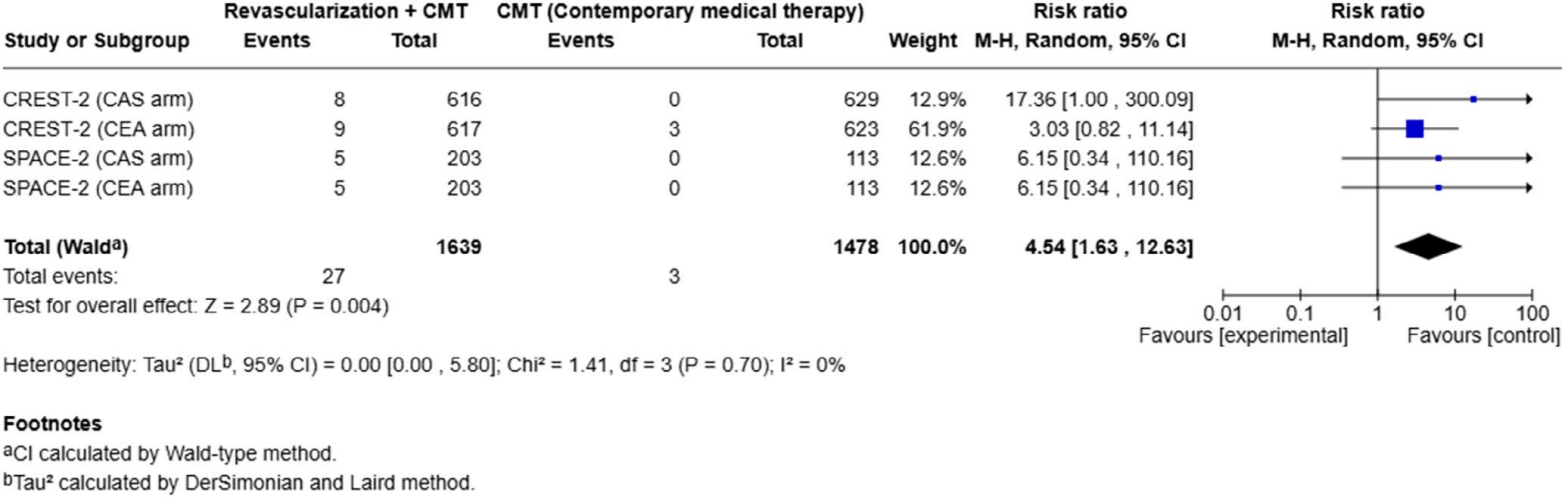
Forest Plot—Periprocedural Complications (Excluding ECST‐2—Sensitivity Analysis). Forest plot of periprocedural complications (30‐day stroke, myocardial infarction, or death) comparing carotid revascularization plus contemporary medical therapy (CMT) versus CMT alone, excluding ECST‐2 (sensitivity analysis). Data from four study arms across two trials: Carotid Revascularization and Medical Management for Asymptomatic Carotid Stenosis Trial (CREST‐2) and Stent‐Protected Angioplasty versus Carotid Endarterectomy in Asymptomatic Patients (SPACE‐2). The pooled risk ratio (RR) was 4.54 (95% CI 1.63–12.63; *p* = 0.004), with no heterogeneity (*I* ^2^ = 0%). Squares represent study‐specific RRs with size proportional to weight; horizontal lines represent 95% confidence intervals; diamond represents pooled estimate.

After excluding ECST‐2, revascularization significantly increased the risk of periprocedural complications (RR 4.54; 95% CI 1.63–12.63; *p* = 0.004; *I* ^2^ = 0%) (Figure 6). Absolute risk increase was 1.45% (number needed to harm = 69).

### 3.4 Subgroup Analysis on the Basis of Treatment Modality

#### 3.4.1 Long‐Term Ipsilateral Stroke

**FIGURE 7:**
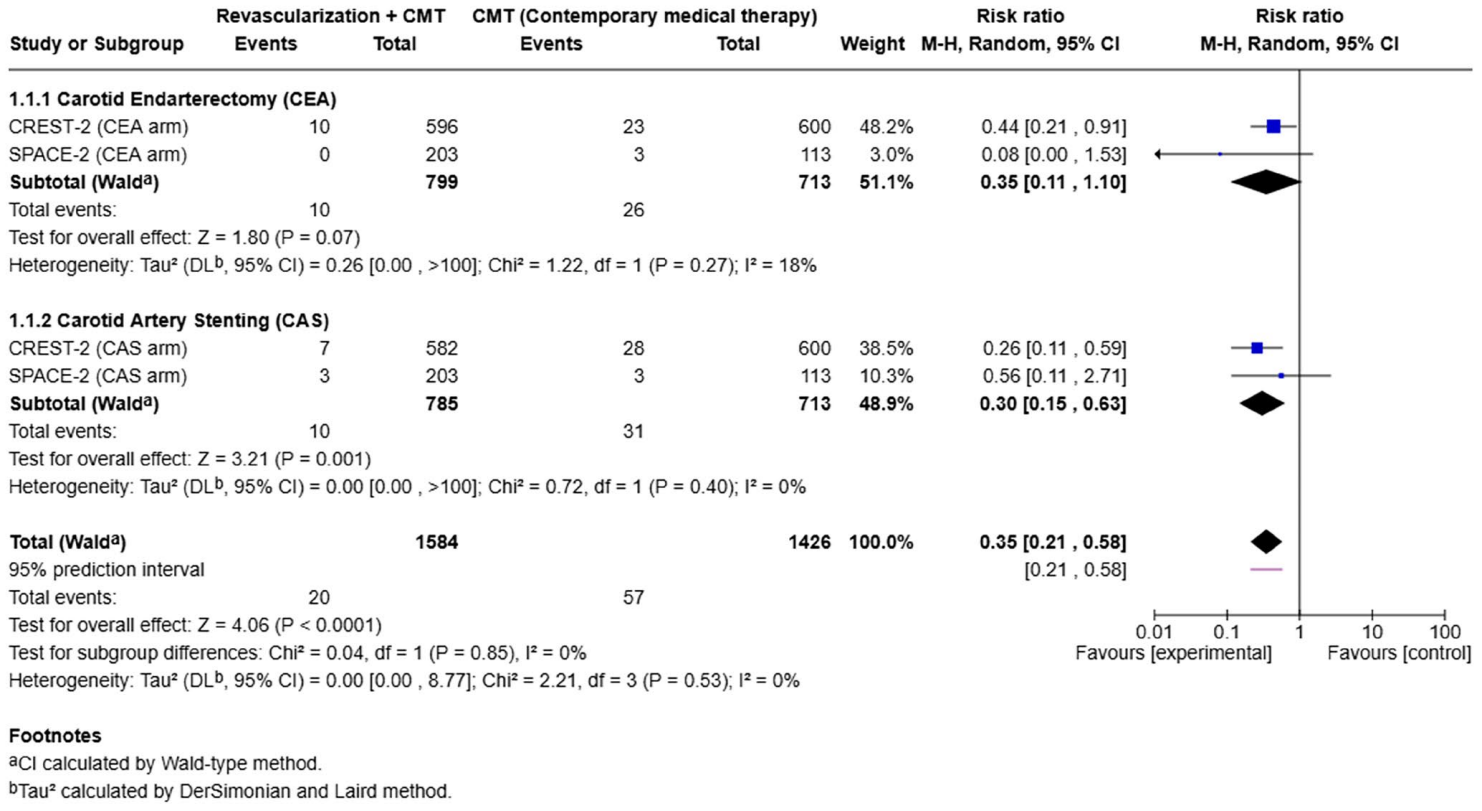
Forest Plot—Subgroup Analysis by Revascularization Modality for Long‐term Ipsilateral Stroke. Forest plot comparing carotid endarterectomy (CEA) versus carotid artery stenting (CAS) for long‐term ipsilateral stroke. Data from four study arms across two trials: CREST‐2 and SPACE‐2. Pooled risk ratios (RR) with 95% confidence intervals (CI) were: CEA subgroup, RR 0.35 (95% CI 0.11–1.10; *p* = 0.07; *I* ^2^ = 18%); CAS subgroup, RR 0.30 (95% CI 0.15–0.63; *p* = 0.001; *I* ^2^ = 0%). Test for subgroup differences: *χ* ^2^ = 0.04, *p* = 0.85, *I* ^2^ = 0%, indicating no significant difference between modalities. CAS, carotid artery stenting; CEA, carotid endarterectomy; CI, confidence interval; RR, risk ratio.

For the CEA subgroup (CREST‐2 CEA + SPACE‐2 CEA), the pooled RR was 0.35 (95% CI 0.11–1.10; *p* = 0.07; *I* ^2^ = 18%), showing a nonsignificant trend toward benefit. For the CAS subgroup (CREST‐2 CAS + SPACE‐2 CAS), the pooled RR was 0.30 (95% CI 0.15–0.63; *p* = 0.001; *I* ^2^ = 0%), showing significant benefit. The test for subgroup differences was not significant (*p* = 0.85), indicating no differential treatment effect between modalities (Figure 7).

#### 3.4.2 Periprocedural Complications

**FIGURE 8:**
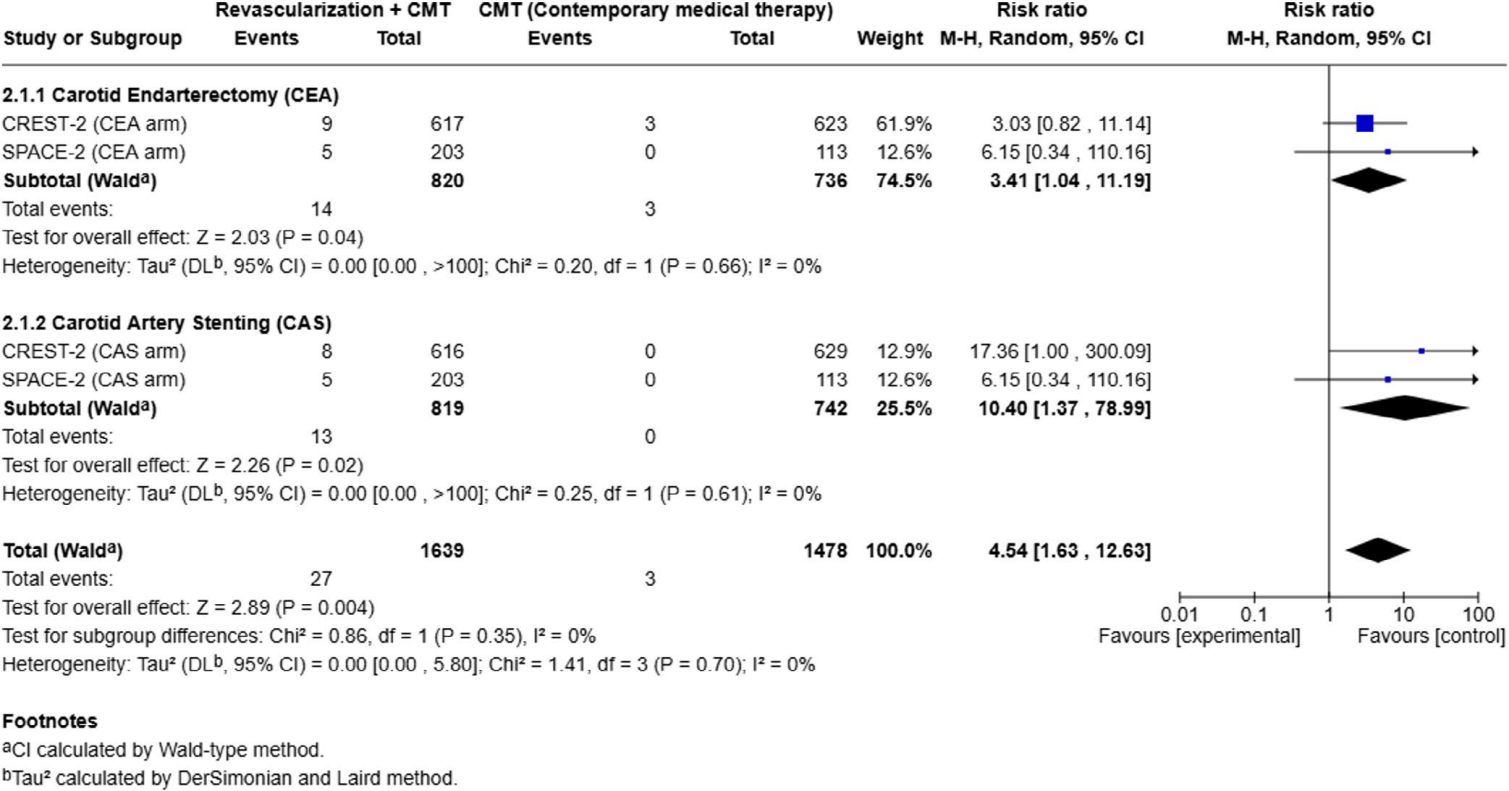
Forest Plot—Subgroup Analysis by Revascularization Modality for Periprocedural Complications. Forest plot comparing carotid endarterectomy (CEA) versus carotid artery stenting (CAS) for periprocedural complications (30‐day stroke, myocardial infarction, or death). Data from four study arms across two trials: CREST‐2 and SPACE‐2. Pooled risk ratios (RR) with 95% confidence intervals (CI) were: CEA subgroup, RR 3.41 (95% CI 1.04–11.19; *p* = 0.04; *I* ^2^ = 0%); CAS subgroup, RR 10.40 (95% CI 1.37–78.99; *p* = 0.02; *I* ^2^ = 0%). Test for subgroup differences: *χ* ^2^ = 0.78, *p* = 0.35, *I* ^2^ = 0%, indicating no significant difference in risk between modalities. CAS, carotid artery stenting; CEA, carotid endarterectomy; CI, confidence interval; RR, risk ratio.

For the CEA subgroup (CREST‐2 CEA + SPACE‐2 CEA), the pooled RR was 3.41 (95% CI 1.04–11.19; *p* = 0.04; *I* ^2^ = 0%), showing significant harm. For the CAS subgroup (CREST‐2 CAS + SPACE‐2 CAS), the pooled RR was 10.40 (95% CI 1.37–78.99; *p* = 0.02; *I* ^2^ = 0%), also showing significant harm. The test for subgroup differences was not significant (*p* = 0.35), indicating no differential risk between modalities (Figure 8).

#### 3.4.3 Sensitivity Analyses

Sensitivity analyses confirmed the robustness of findings after excluding ECST‐2. Using a fixed‐effects model instead of random‐effects yielded similar results for the sensitivity analysis (long‐term stroke: RR 0.35; 95% CI 0.21–0.58; periprocedural: RR 4.54; 95% CI 1.63–12.63). Leave‐one‐out analysis showed no single study disproportionately influenced the pooled estimates after ECST‐2 exclusion.

## 4 Discussion

This meta‐analysis of three contemporary randomized trials (CREST‐2, SPACE‐2, and ECST‐2) provides updated insight into the balance between carotid revascularization and contemporary medical therapy (CMT) in asymptomatic carotid stenosis [12, 13, 14]. The primary pooled analysis including ECST‐2 was statistically nonsignificant and demonstrated substantial heterogeneity, suggesting uncertainty regarding the overall treatment effect. However, sensitivity analyses restricted to purely asymptomatic populations suggested that revascularization may reduce long‐term ipsilateral stroke while increasing periprocedural complications. These findings should therefore be interpreted cautiously and viewed as hypothesis‐generating rather than definitive evidence supporting routine revascularization.

The divergent findings of ECST‐2 likely reflect important methodological and clinical differences from CREST‐2 and SPACE‐2 [14]. Unlike the other trials, ECST‐2 enrolled both asymptomatic patients and symptomatic patients only if they had low‐to‐intermediate predicted stroke risk according to the CAR score. Consequently, the ECST‐2 population may have had substantially lower baseline stroke risk than historical symptomatic carotid stenosis cohorts and potentially lower risk than the populations enrolled in CREST‐2 and SPACE‐2. In addition, ECST‐2 used intensive optimized medical therapy, lower stenosis thresholds, and relatively short follow‐up, all of which may have contributed to the opposite direction of effect and observed heterogeneity [14, 15].

Compared with historical trials such as ACAS and ACST, contemporary medical therapy has substantially lowered the baseline risk of stroke in asymptomatic carotid stenosis [3, 4]. Modern aggressive management with high‐intensity statins, blood pressure control, smoking cessation, and antiplatelet therapy has reduced annual ipsilateral stroke risk to approximately 1% or lower in many patients [7, 8, 9, 10, 11]. Consequently, the absolute benefit achievable with revascularization appears considerably smaller than in earlier eras, narrowing the risk–benefit margin and making routine intervention more difficult to justify [11, 15, 16].

### 4.1 Subgroup Analysis: CEA Vs. CAS

Subgroup analyses suggested numerically different treatment estimates between carotid endarterectomy (CEA) and carotid artery stenting (CAS), although these findings should be interpreted cautiously. CAS demonstrated a statistically significant reduction in long‐term ipsilateral stroke, whereas CEA showed only a nonsignificant trend. However, the number of included studies and outcome events was limited, and the analysis was underpowered to establish superiority or inferiority between modalities [17, 18, 19]. The apparent subgroup differences may therefore reflect statistical instability, differences in event distribution, or random variation rather than true modality‐specific effects.

Both CEA and CAS increased periprocedural complications, without a statistically significant difference between modalities. Procedural expertise, patient anatomy, plaque morphology, and center experience likely influence outcomes substantially and cannot be adequately evaluated within the current dataset [19, 21, 22, 23]. Therefore, these subgroup findings should be considered exploratory and should not be used alone to guide modality selection in clinical practice.

### 4.2 Clinical Implications

The findings of this meta‐analysis support contemporary medical therapy as the foundational treatment strategy for most patients with asymptomatic carotid stenosis [2, 7, 8, 24, 25]. Across all included trials, optimized medical therapy was delivered in highly controlled settings with intensive management of blood pressure, lipid levels, smoking cessation, and antithrombotic therapy. The resulting low stroke rates highlight the effectiveness of modern vascular prevention strategies and reduce the absolute benefit achievable with routine revascularization.

At the same time, these findings should not be generalized to all patients with asymptomatic carotid stenosis. CREST‐2 and SPACE‐2 enrolled carefully selected patients with asymptomatic high‐grade stenosis managed at experienced centers under strict procedural credentialing and intensive follow‐up protocols. ECST‐2 further selected lower‐risk patients using CAR‐score stratification [12, 13, 14]. Therefore, the results are most applicable to carefully selected patients managed in specialized centers with optimized contemporary medical therapy.

Revascularization may still be reasonable in selected higher‐risk patients despite optimized medical therapy [6, 11]. Potential candidates may include those with high‐risk plaque characteristics, rapidly progressive stenosis, silent cerebral infarction, or other markers associated with increased future stroke risk [26, 27, 28]. However, these features were not systematically used as enrollment criteria in CREST‐2, SPACE‐2, or ECST‐2 and therefore remain hypothesis‐generating rather than validated treatment‐selection markers. Shared decision‐making remains essential, particularly given the trade‐off between modest long‐term stroke reduction and increased periprocedural risk [1, 29, 30, 31, 32].

### 4.3 Strengths and Limitations

This study has several strengths, including restriction to contemporary randomized trials, separation of long‐term stroke outcomes from periprocedural complications, prespecified sensitivity analyses, and evaluation of modality‐specific subgroups. In addition, sensitivity analyses using split‐control methodology for SPACE‐2 yielded directionally similar findings, supporting the robustness of the overall signal despite reduced statistical precision.

Several limitations should also be acknowledged. First, the primary pooled analysis demonstrated substantial heterogeneity driven largely by ECST‐2, which enrolled a methodologically distinct mixed low‐risk population [14]. Second, SPACE‐2 terminated early and had limited statistical power [13]. Third, differences in stenosis thresholds, follow‐up duration, and patient selection criteria across studies may limit comparability [12, 13, 14]. Fourth, the overall number of trials and outcome events was small, limiting statistical precision and the reliability of subgroup analyses. Finally, the low event rates observed with optimized medical therapy reduce the ability to detect modest absolute benefits from revascularization.

### 4.4 Future Directions

Future research should focus on improving risk stratification to better identify patients who may derive meaningful benefit from revascularization despite optimized medical therapy [11, 26, 27, 28]. Advanced plaque imaging, transcranial Doppler microembolic monitoring, and circulating biomarkers may help identify higher‐risk subgroups in future studies [33, 34, 35, 36]. Longer‐term follow‐up from contemporary randomized trials will also be important to determine whether any delayed benefit from revascularization emerges beyond the currently available follow‐up periods [12, 13, 14].

## 5 Conclusion

In this pooled analysis of contemporary randomized trials, the primary analysis including ECST‐2 was inconclusive and demonstrated substantial heterogeneity. Sensitivity analyses restricted to purely asymptomatic populations suggested that revascularization may reduce long‐term ipsilateral stroke but at the cost of increased periprocedural complications. These findings should be interpreted cautiously given the limited number of trials, low event rates, and sensitivity of the pooled estimates to study selection and analytic assumptions. Contemporary medical therapy remains the foundation of management for asymptomatic carotid stenosis, while revascularization should be reserved for carefully selected patients after individualized risk assessment and shared decision‐making.

## Author Contributions

Aasim Ali and Waqar Ahmed Cheema conceived the study, designed the protocol, developed the search strategy, supervised data extraction, performed the statistical analysis, interpreted the results, and drafted the initial manuscript. Fiza Nisar, Muhammad Shamoon, Sameen Sarfaraz, Ahmad Butt, Muhammad Abdullah Ali, Armaghana Abdullah, Muhammad Asad Shabbir, Muhammad Talha, Hammad Azam, Aimen Anwaar Pannun, Usama Saleem, and Anousha Tanveer contributed to literature screening, eligibility assessment, data extraction, risk‐of‐bias assessment, verification of extracted outcomes, and preparation of tables and figures. Mukesh Sharma supervised the project, resolved methodological disagreements, critically reviewed the manuscript for important intellectual content, and approved the final version for submission. All authors reviewed and edited the manuscript, approved the final submitted version, and agree to be accountable for all aspects of the work.

## Funding

The authors have nothing to report.

## Conflicts of Interest

The authors declare no conflicts of interest.

## Supporting information


**Table S1:** Comprehensive Search Strategy.
**Table S2:** GRADE Evidence Profile Table.
**Figure S1:** Split group of SPACE 2 analysis.

## Data Availability

All data analyzed in this systematic review and meta‐analysis were extracted from previously published studies and publicly available trial reports cited in the manuscript. No new primary data were generated. The extracted dataset used for the analysis is available from the corresponding author upon reasonable request.
