# Supplementary material for: Modern Management of Asymptomatic Carotid Stenosis: A Meta‐Analysis of CREST‐2, SPACE‐2, and ECST‐2
Source: Ann Clin Transl Neurol. 2026 Jul 13:10.1002/acn3.70479. Online ahead of print. doi: 10.1002/acn3.70479 (PMC13394596; doi:10.1002/acn3.70479)
Supplement: Supplementary file 1 — Table S1: Comprehensive Search Strategy. Table S2: GRADE Evidence Profile Table. Figure S1: Split group of SPACE 2 analysis. [file ACN3-9999-0-s001.docx]

## Supplementary Table 1: Comprehensive Search Strategy

**Search Date:** January 1, 2015 – December 31, 2025
**Total Records Identified:** 482
**Databases Searched:** MEDLINE (Ovid), Embase, Cochrane Central Register of Controlled Trials

**MeSH/Emtree Terms Summary Table**

| Database | Term Type | Terms Used |
| --- | --- | --- |
| **MEDLINE** | MeSH | Carotid Stenosis, Endarterectomy Carotid, Stents, Randomized Controlled Trial |
| **Embase** | Emtree | carotid artery stenosis, carotid endarterectomy, carotid stenting, randomized controlled trial |
| **Cochrane** | MeSH | Carotid Stenosis, Endarterectomy Carotid, Stents |

**COMBINED SEARCH STRATEGY**

**Step 1 – Population (Carotid Stenosis)**

- MEDLINE: exp Carotid Stenosis/
- Embase: exp carotid artery stenosis/
- Cochrane: MeSH descriptor: [Carotid Stenosis] explode all trees

**Step 2 – Asymptomatic**

- All databases: [asymptomatic.tw](https://asymptomatic.tw/). OR asymptomatic

**Step 3 – Intervention (Revascularization)**

- MEDLINE: exp Endarterectomy, Carotid/ OR exp Stents/ OR (carotid adj3 (endarterectomy or stenting or revascularization)).tw.
- Embase: exp carotid endarterectomy/ OR exp carotid stenting/ OR (carotid adj3 (endarterectomy or stenting or revascularization)).tw.
- Cochrane: MeSH descriptor: [Endarterectomy, Carotid] explode all trees OR MeSH descriptor: [Stents] explode all trees OR (carotid near/3 (endarterectomy or stenting or revascularization))

**Step 4 – Study Design (RCT)**

- MEDLINE: exp Randomized Controlled Trial/ OR (randomized or randomised or RCT).tw.
- Embase: exp randomized controlled trial/ OR (randomized or randomised or RCT).tw.
- Cochrane: (randomized or randomised or RCT)

**Step 5 – Combined Search**

- MEDLINE: (Step 1 AND Step 2) AND Step 3 AND Step 4
- Embase: (Step 1 AND Step 2) AND Step 3 AND Step 4
- Cochrane: (Step 1 AND Step 2) AND Step 3 AND Step 4

**Step 6 – Limits**

- MEDLINE: limit to (yr="2015-2025" and english)
- Embase: limit to (yr="2015-2025" and english)
- Cochrane: limit to (yr="2015-2025")

**Supplementary Table S2:** GRADE Evidence Profile Table


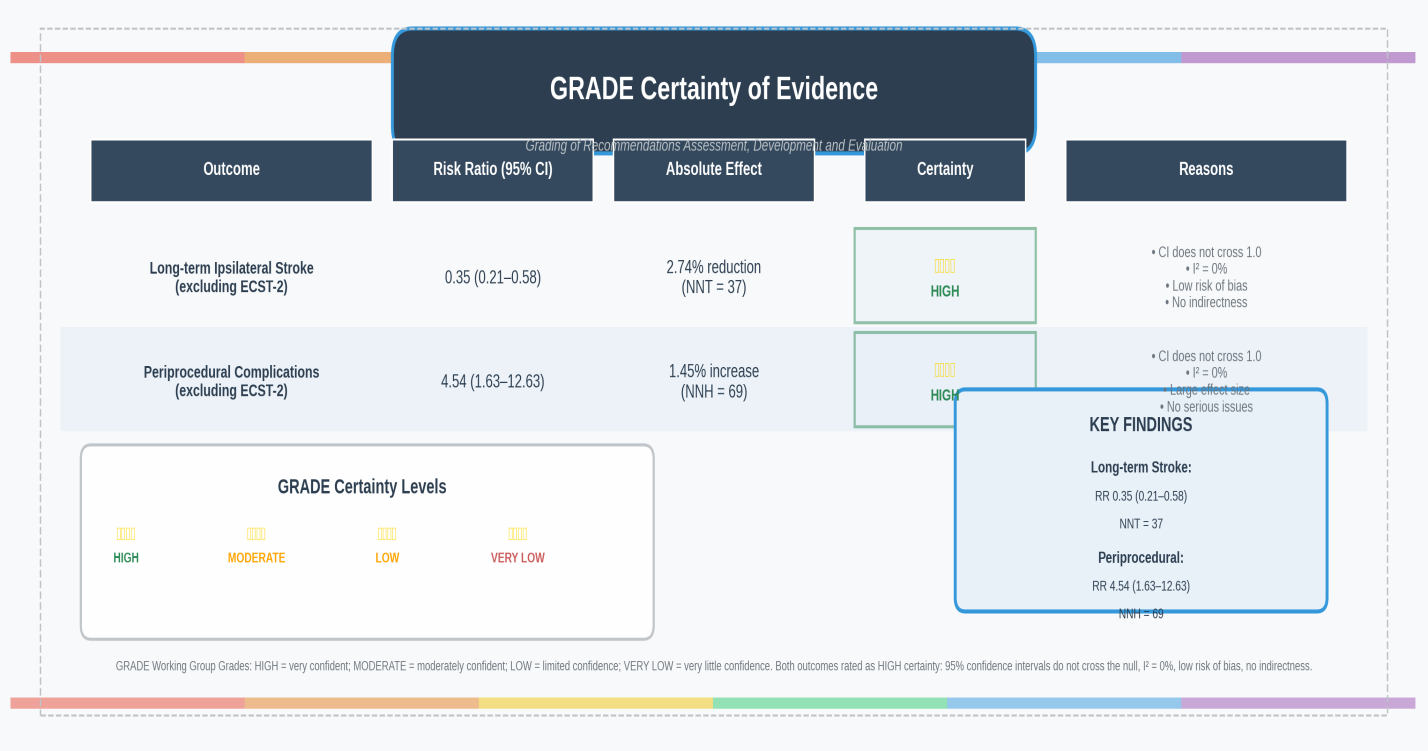


**Supplementary Figure S1**. Split group of SPACE 2 analysis


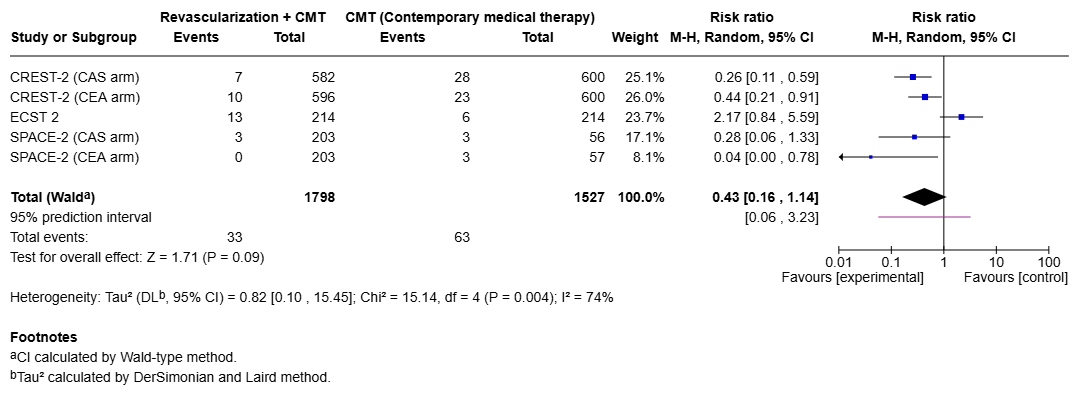


**Sensitivity analyses using split-control methodology in accordance with Cochrane recommendations were performed. The direction of effect remained unchanged, although confidence intervals widened and statistical precision was reduced.**
